# Sotatercept in Children with Pulmonary Hypertension—A Narrative Review

**DOI:** 10.3390/children13040465

**Published:** 2026-03-28

**Authors:** Johanna Schulz, Veronika C. Stark, Lars Harbaum, Rainer Kozlik-Feldmann, Thomas S. Mir, Fridrike Stute, Jakob Olfe

**Affiliations:** 1 Pediatric Cardiology, Children’s Heart Clinic, University Heart & Vascular Center Hamburg, Martinistraße 52, 20246 Hamburg, Germanyj.olfe@uke.de (J.O.); 2 Division of Respiratory Medicine, Department of Internal Medicine II, University Medical Centre Hamburg-Eppendorf Hamburg, Martinistraße 52, 20246 Hamburg, Germany

**Keywords:** pediatric pulmonary hypertension, sotatercept, disease-modifying therapy, persistent pulmonary hypertension of the newborn, children, pulmonary vascular remodeling

## Abstract

**Highlights:**

**What are the main findings?**

- Sotatercept demonstrates disease-modifying effects in adult PAH trials by reducing PVR, improving exercise capacity and right ventricular function via TGF-β pathway modulation, with preclinical evidence of vascular remodeling reversal.
- Extrapolation to children is supported by shared pathobiology but limited by etiological differences and a lack of pediatric RCTs, with MOONBEAM being the first targeted study.

**What are the implications of the main findings?**

- Sotatercept could transform pediatric PAH management as an add-on to vasodilators, potentially improving survival and quality of life if efficacy translates.
- There is an urgent need for long-term pediatric safety data on activin inhibition risks (tumorigenesis, puberty, fertility, bone growth) to inform regulatory approval and clinical protocols.

**Abstract:**

Background/Objectives: Pulmonary arterial hypertension (PAH) is a rare but life-threatening disease that presents particular therapeutic challenges in children. It is characterized by pulmonary vasoconstriction and vascular remodeling, leading to right ventricular strain and eventually right heart failure. Although advances in pharmacotherapy have improved outcomes, treatment options remain limited. This review aims to evaluate the potential role of sotatercept, a novel fusion protein recently approved for adult PAH, and to assess the translatability of adult data to the pediatric population. Methods: A narrative synthesis of preclinical studies and randomized controlled trials was conducted to summarize the current evidence on sotatercept. In addition, pathophysiological, developmental, and therapeutic differences between adult and pediatric PAH were critically examined to assess relevance and applicability to younger patients. Results: Clinical trials in adults (PULSAR, STELLAR, ZENITH, HYPERION) confirm sotatercept’s efficacy on background therapy, with significant reductions in pulmonary vascular resistance, improvements in 6 min walk distance, enhanced right ventricular function, and risk reductions in clinical worsening events. However, extrapolation to pediatric PAH faces challenges including etiological differences (e.g., PAH-CHD predominance, PPHN in infants), age-inappropriate endpoints (e.g., 6MWD infeasible in young children), variable growth-related pharmacokinetics, and compensatory RV physiology delaying overt failure. Safety concerns are manageable in adults but raise pediatric-specific alarms: activin inhibition’s theoretical tumorigenic potential (dual tumor suppressor/promoter role), pubertal/fertility disruption (FSH suppression, gonadal maturation delay), and skeletal growth interference—unproven clinically yet demanding long-term monitoring. The ongoing MOONBEAM trial will provide initial pharmacokinetic/safety data in children. Conclusions: Sotatercept represents a promising, first-in-class therapeutic option for PAH with the potential to transform disease management. Nevertheless, dedicated pediatric studies are crucial to confirm safety, efficacy, and appropriate dosing and to define its role in the long-term treatment of children with PAH.

## 1. Introduction

Pulmonary hypertension (PH) comprises a variety of serious diseases and has a prevalence of approximately 1% of the global population [1]. All age groups can be affected. PH is defined by an elevated mean pulmonary artery pressure (mPAP) of >20 mmHg in patients older than 3 months. Due to its diverse causes, PH is divided into five main groups based on etiology, clinical presentation, and hemodynamic parameters.

Pulmonary arterial hypertension (PAH) is a rare but clinically important subtype [2]. It is the most common form of PH in childhood and is estimated to have an incidence of 2–2.2 cases per 1 million children [3]. PAH is divided into several subgroups, including idiopathic PAH (IPAH), hereditary PAH (HPAH), and persistent pulmonary hypertension of the newborn (PPHN). Together with PAH associated with congenital heart disease (PAH-CHD), these forms represent the majority of cases in children [4]. PAH is one of the precapillary forms of PH and is characterized by pulmonary vasoconstriction and vascular remodeling in the pulmonary arteries. Histopathologically, it is reflected by intimal proliferation, medial hypertrophy, increased inflammation, the occasional occurrence of plexiform lesions, and local thrombotic events [5]. These pathological changes lead to a progressive narrowing of the vascular lumen accompanied by an increase in pulmonary vascular resistance (PVR) and mean pulmonary arterial pressure (mPAP), which causes increasing stress on the right ventricle (RV) and results in right heart dilation and hypertrophy, right heart failure, and premature death [6]. However, the exact etiology of PAH pathogenesis is complex and based on genetic, epigenetic, and environmental factors. The genetic component shows great heterogeneity, but current research highlights the role of altered signal transduction by members of the transforming growth factor-β (TGF-β) family, such as bone morphogenetic protein receptor 2 (BMPR2), in vascular remodeling [7]. BMPR2 mutations are the most common genetic finding and occur in up to 20% of IPAH cases and up to 70% of HPAH cases [8].

The symptoms of PAH are nonspecific and are largely due to right ventricular dysfunction. Typical symptoms in adults and adolescents include rapid fatigue, exertional dyspnea, (pre-)syncope, and even right heart failure and cyanosis [2]. In contrast, infants and young children present with poor appetite, failure to thrive and irritability [4]. The severity of pulmonary hypertension is classified into four functional groups according to the World Health Organization (WHO) functional class, based on clinical symptoms. Along with NT-proBNP levels and 6 min walk distance (6MWD), the WHO functional class is one of the most powerful predictors of patient survival [9]. At the time of diagnosis, children with PAH often appear clinically more stable than adults, despite the disease being equally severe. This is reflected in better preserved cardiac output, lower right atrial pressure, and higher mixed venous oxygen saturation. It is assumed that pediatric patients have a greater physiological capacity to compensate for the increased RV load. This compensatory ability may contribute to the later onset of right heart failure typically observed in children [10].

In addition to clinical examination, diagnostic imaging plays an important role in the diagnosis of PAH. Basic diagnostics should consist of echocardiography, ECG, chest X-ray, and pulse oximetry. To exclude other causes, lung function tests and functional stress tests are necessary. These can also be used to assess mortality risk as a diagnosis and during follow-up. CT angiography and ventilation/perfusion scintigraphy (VQ scintigraphy) are also mandatory imaging procedures for confirming the diagnosis of IPAH/HPAH by excluding other forms of PH associated with structural changes in the lungs and thromboembolism. However, the gold standard for the diagnosis and classification of PAH remains right heart catheterization, as it allows for a very accurate assessment of hemodynamic conditions. Characteristic results for PAH are mPAP > 20 mmHg, PVR > 2 Wood units (WU), and pulmonary artery wedge pressure (PAWP) ≤ 15 mmHg. In addition, an assessment of acute pulmonary vasoreactivity during catheterization is essential to select patients for specific treatment regimens [2].

Given that PAH is currently a chronic, progressive, non-curable disease, the goal of treatment is limited to stabilizing the disease. Drug-based therapy is currently the standard treatment. It includes typical substance groups such as calcium channel blockers, phosphodiesterase-5 (PDE5) inhibitors, prostacyclin analogs, endothelin receptor antagonists, and soluble guanylate cyclase stimulators [2]. They improve physical activity and slow the progression of PAH. However, they primarily work through symptomatic vasodilation and have only a limited effect on the underlying pathomechanism, vascular and right ventricular remodeling [11]. Currently, only two active substances are approved by the U.S. Food and Drug Administration (FDA) for the treatment of pediatric PAH: sildenafil, a PDE5 inhibitor, and bosentan, an endothelin receptor antagonist. In contrast, within the EU, four additional drugs have been approved by the European Medicines Agency (EMA): ambrisentan and macitentan, additional endothelin receptor antagonists; tadalafil, another PDE5 inhibitor; and riociguat, representing a new class of soluble guanylate cyclase stimulators [12]. Prostacyclin derivatives and calcium channel blockers are also used off-label [4]. In international registries, up to 80% of pediatric patients receive at least one PAH-targeted therapy [13]. Despite improvements in mortality rates over recent decades, the 5-year survival rate for children receiving adequate treatment is 72% [14].

Sotatercept represents a promising new treatment option for PAH. In contrast to the currently established therapy, it works through a completely different mechanism and specifically interferes with the remodeling processes of PAH [15]. The following work aims to explore the potential role of sotatercept as a therapeutic option for the treatment of pediatric PAH. Emphasis is placed on elucidating the mechanism of action and efficacy of sotatercept, as well as highlighting the similarities and differences in PAH between adults and children.

## 2. Materials and Methods

**Figure 1:**
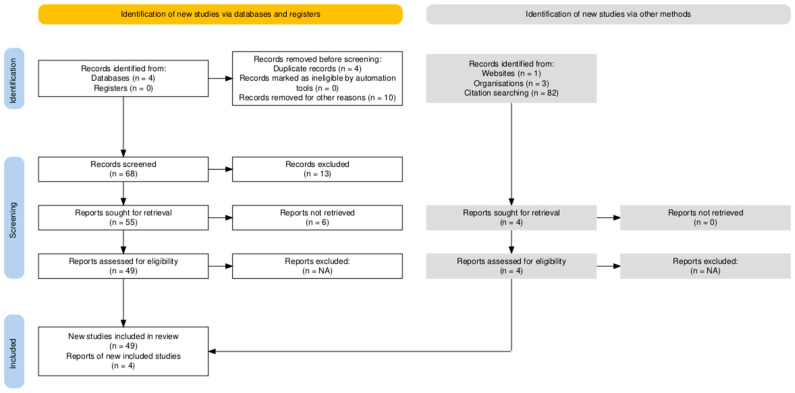
An adapted search flow diagram showing the different phases of the review process [16].

This work is based on a targeted literature search conducted in the medical databases PubMed, Embase, Scopus and Web of Science to present the current state of knowledge regarding the use of sotatercept in PAH, with a particular focus on its potential relevance in pediatrics. The search was carried out between January and November 2025. The various search terms used were: “sotatercept”, “pulmonary arterial hypertension”, “PAH”, “children”, persistent pulmonary hypertension in the newborn”, “PPHN”, “Activin A” and “paediatric”. Clinical randomized controlled trials, meta-analyses, reviews and international guidelines published up to and including October 2025 in the English language were eligible for inclusion. U.S. Food and Drug Administration, European Medicines Agency and pharmaceutical company data sheets were included. A record was excluded when it was a shorter and less detailed version of a review, its written language was not English or data could not be extracted (Figure 1). Screening was performed by two reviewers (JS and JO). Due to the lack of completed pediatric studies on sotatercept, studies involving adult patient populations were primarily considered. The scope of this review is a narrative synthesis, not a systematic review, to describe current evidence according to landmark RCTs, pivotal trials and guidelines.

## 3. Results

### 3.1. Mechanism of Action

Sotatercept represents a promising therapeutic option for PAH by specifically targeting the underlying pathophysiological mechanisms. In 2024, it became the first-in-class agent approved by the FDA and EMA for the treatment of PAH patients receiving background therapy. The following evidence from preclinical and clinical studies shows its mechanism of action and potential therapeutic benefits.

Sotatercept is a fusion protein that functions as a ligand trap. It consists of the Fc fragment of a human immunoglobulin (IgG1) linked to the extracellular domain of the activin-binding receptor type IIA (ACTRIIA). This domain binds and neutralizes specific ligands of the TGF-β superfamily [17]. The dysregulation of these ligands plays a crucial role in the development of PAH. It leads to an imbalance in SMAD signaling pathways in pulmonary endothelial cells (ECs) and vascular smooth muscle cells (SMCs). More specifically, it causes the overactivation of pro-proliferative SMAD2/3 and suppression of anti-proliferative SMAD1/5/8 transcription factors. A mutation in the BMPR2 gene further amplifies this mechanism. A reduced BMPR2 level leads to the decreased activation of the anti-proliferative SMAD1/5/8 transcription factors. This results in a reduction in the apoptosis rate of SMCs and the loss of EC quiescence, leading to hyper-proliferation with the muscularization of distal pulmonary arteries/arterioles, as well as vascular fibrosis. The disrupted regulation of the SMAD1/5/8 signaling pathway also leads to the increased production of ligands of the TGF-β family, such as activins A and B and growth differentiation factors (GDFs) 8 and 11. These ligands further enhance the process of hyper-proliferation by activating the pro-proliferative SMAD2/3 signaling pathway [7].

Sotatercept uses a therapeutic approach that aims to restore the balance of SMAD signaling. Its ACTRIIA-Fc domain binds ligands of the TGF-β superfamily, in particular activin A, activin B, and GDFs 8 and 1, thereby reducing their availability to bind and activate respective receptors. Under normal conditions, these receptors form transmembrane complexes consisting of two type I (e.g., BMPRII, ALK1) and two type II receptors (e.g., ActRIIA, ActRIIB, TGFβRII) with serine/threonine kinase activity. When ligands engage these receptors, the type II receptors phosphorylate the type I receptors, initiating downstream signaling cascades [18]. By neutralizing the ligands, sotatercept inhibits pro-proliferative SMAD2/3 signaling while indirectly activating the anti-proliferative SMAD1/5/8 signaling pathway [17]. Experimental PAH models have shown that this causes a reduction and even reversal of vascular remodeling. The anti-remodeling effects are primarily associated with the inhibition of SMAD2/3 activation and thus reduced vascular proliferation, as well as an increased rate of apoptosis. In addition, treatment with sotatercept shows a reduction in macrophage infiltration and activation in lung tissue. This leads to reduced inflammation, which is considered an important amplifier of PAH pathogenesis [19]. In preclinical PAH models, treatment with ACTRIIA-Fc also showed an improvement in cardio-pulmonary function. Among other findings, there was a significant reduction in right ventricular systolic pressure (RVSP), a decrease in the total pulmonary resistance index (TPRI), and a reduction in right ventricular hypertrophy. Compared to classic vasodilators, sotatercept showed a superior effect in this regard [17].

### 3.2. Current State of Evidence

The efficacy of sotatercept has also been proven in several clinical trials (Table 1). However, the pharmacokinetic properties of sotatercept should first be explained in more detail. Sotatercept has a bioavailability of 66% after subcutaneous application. At a dosage of 0.7 mg/kg subcutaneously at three-week intervals, a maximum concentration of 9.7 µg/mL is reached 7 days after injection. The half-life is approximately 24 days, with metabolism occurring through general protein degradation processes. A steady state is reached after approximately 15 weeks with the appropriate dosage [20].

The PULSAR study, published in the *New England Journal of Medicine* in 2021, investigated the efficacy and safety of sotatercept for the treatment of PAH in patients already on background therapy. In this multicenter, randomized, double-blind Phase 2 study, 106 patients with confirmed PAH were randomized 1:1:1 to receive either placebo or sotatercept at a dose of 0.3 mg/kg or 0.7 mg/kg subcutaneously every 3 weeks. Inclusion criteria comprised an age of over 18 years, a confirmed diagnosis of PAH in WHO functional class II/III, and a stable background therapy for at least 90 days. Patients with specific PAH subtypes, including porto-pulmonary disease, schistosomiasis, HIV-associated PAH, or pulmonary veno-occlusive disease, were excluded. Demographic aspects in the three subgroups barely differed. This study included a 24-week treatment phase followed by an 18-month active extension phase. The primary endpoint was the change in PVR from baseline to week 24. The results showed a significant reduction in PVR in the sotatercept groups compared to the placebo group. The least squares mean (LSM) changed by −162.2 dyn·s·cm^−5^ in the sotatercept group (0.3 mg/kg), −255.9 dyn·s·cm^−5^ in the sotatercept group (0.7 mg/kg), and −16.4 dyn·s·cm^−5^ in the placebo group. The LSM difference was −145.8 dyn·s·cm^−5^ (95% confidence interval (CI), −241.0 to −50.6; *p* = 0.003) between the sotatercept (0.3 mg/kg) and placebo groups and 239.5 dyn·s·cm^−5^ (95% CI, −329.3 to −149.7; *p* < 0.001) between the sotatercept (0.7 mg/kg) and placebo groups. There was also an improvement in the 6 min walk distance (6MWD), one of the most important secondary endpoints. In week 24, the LSM difference between the sotatercept (0.3 mg/kg) and placebo groups was +29.4 m (95% CI, 3.8 to 55.0). The LSM difference between the sotatercept group (0.7 mg/kg) and the placebo group was +21.4 m (95% CI, −2.8 to 45.7). In addition, sotatercept was associated with a reduction in N-terminal pro-B-type natriuretic peptide (NT-proBNP) levels. One patient in the sotatercept group (0.7 mg/kg) died of cardiac arrest. In summary, treatment with sotatercept resulted in a significant reduction in PVR in patients receiving background therapy for PAH [21].

The STELLAR study, published in the *New England Journal of Medicine* in 2023, confirmed the beneficial effect. This is a multicenter, double-blind, randomized, placebo-controlled Phase 3 study investigating the efficacy and safety of sotatercept in PAH. A total of 323 adult patients with PAH on background therapy were enrolled and randomized 1:1 to receive either sotatercept or placebo. Inclusion criteria included age > 18 years, symptomatic PAH of WHO functional class II/III, a baseline PVR of ≥400 dyn·s·cm^−5^ (5 WU), and a 6MWD ≥ 150 m and ≤500 m. In addition, stable background therapy for PAH for at least 90 days prior to enrolment was required. Excluded were patients with PAH associated with HIV infection, portal hypertension, schistosomiasis, or pulmonary veno-occlusive disease. Treatment consisted of the subcutaneous injection of sotatercept or placebo every 3 weeks in addition to background therapy (mono-, dual, or triple therapy). Sotatercept was initially given at a starting dose of 0.3 mg/kg, with a target dose of 0.7 mg/kg from day 21 onwards. The patient group was predominantly female (79.3%) with an average age of 47.9 ± 14.8 years with a mean length of time since diagnosis and treatment for PAH of 8.8 years. The most common forms of PAH were idiopathic (58.5%) and hereditary PAH (18.3%). Additionally, patients with PAH associated with connective tissue diseases (14.9%), PAH associated with corrected congenital shunts (5%), or drug- or toxin-induced PAH (3.4%) were also included. Approximately half of the patients were in WHO functional class II, the other half in class III. The majority (61.3%) received triple therapy as background treatment, including continuous prostacyclin infusion therapy in around 40%, indicating a heavily pretreated, prevalent PAH population. The primary endpoint was the change in 6MWD after 24 weeks compared to baseline. The mean change in 6MWD was +34.4 m (95% CI, 33.0 to 35.5 m) in the sotatercept group, compared with +1.0 m (95% CI, −0.3 to 3.5 m) in the placebo group. Sotatercept treatment resulted in a significantly greater improvement in 6MWD compared with placebo, with a mean between-group difference of +40.8 m (95% CI, 27.5 to 54.1 m; *p* < 0.001). Significant improvement was also seen in the sotatercept group compared to the placebo group in eight of nine secondary endpoints, including NT-proBNP levels, PVR, WHO functional class, mortality risk (French Risk Score), and parts of the PAH-SYMPACT quality of life and symptom questionnaire [22]. In an echocardiography sub-study of the STELLAR trial, sotatercept also demonstrated substantial improvements in pulmonary artery to right ventricle coupling and right heart function [23].

In the SPECTRA study from 2024, Waxman et al. demonstrated that sotatercept therapy leads to a significant increase in the maximum oxygen uptake in cardio-pulmonary exercise testing (CPET). It was a multicenter, single-arm, open-label, exploratory Phase 2a study in adults with WHO functional class III PAH receiving a combination background therapy. A total of 21 patients were included and received subcutaneous sotatercept every 3 weeks during the 24-week treatment phase, with a starting dose of 0.3 mg/kg and a target dose of 0.7 mg/kg. The primary endpoint was the change in peak oxygen uptake from baseline to week 24. A significant improvement of 102.74 mL/min (95% CI, 27.72 to 177.76, *p* = 0.0097) was demonstrated. Improvements in secondary endpoints such as 6MWD, the resting and peak exercise hemodynamics, and RV function on cardiac MRI were also observed. SPECTRA was the first prospective study to show that sotatercept therapy leads to a reduction in end-diastolic volume and RV mass using gold standard MRI. Both endpoints indicate the reversibility of RV remodeling [24].

The ZENITH study, published in the *New England Journal of Medicine* in 2025, was the first to investigate the efficacy of sotatercept in patients with severe PAH at high mortality risk. It is a multicenter, double-blind, randomized Phase 3 study. Enrolled were 172 patients with WHO functional class III/IV and PVR ≥ 400 dyn·s·cm^−5^ who were receiving maximum background therapy and had a high 1-year mortality risk as indicated by a REVEAL Lite 2 risk score of 9 points or higher. Patients in the trial got either sotatercept or a placebo every 3 weeks in addition to their current treatment. The main outcome was defined as a composite endpoint, the first occurrence of death from any cause, lung transplant, or hospitalization for more than 24 h due to worsening PAH. In the sotatercept group, at least one of these events occurred in 15 patients (17.4%) and in the placebo group in 47 patients (54.7%). This is a significant risk reduction of 76% (hazard ratio 0.24; 95% CI, 0.13 to 0.43, *p* < 0.001). This study was stopped early because the sotatercept group was significantly superior, and the continuation of the placebo arm was no longer ethically justified [25]. The rationale for initiating sotatercept in high-risk patients is supported by two recent case series using continuous remote hemodynamic monitoring. Following the first 0.3 mg/kg dose, a rapid improvement was observed, including an approximate 6 to 8 mmHg reduction in mPAP and an equivalent reduction in RV pressures, indicating that sotatercept confers the rapid unloading of the right ventricle [26,27].

The HYPERION study, published in 2025 in the *New England Journal of Medicine*, investigated the efficacy and safety of sotatercept in adults with newly diagnosed PAH (within the first year) at intermediate or high risk (a REVEAL Lite 2 risk score ≥ 6 or a COMPERA 2.0 risk score ≥ 2). This multicenter, double-blind, randomized, placebo-controlled Phase 3 study enrolled 320 patients, who received either sotatercept or placebo every 3 weeks in addition to stable background therapy. Sotatercept was administered subcutaneously at a starting dose of 0.3 mg/kg, with a target dose of 0.7 mg/kg from day 21 onward. The primary composite endpoint was clinical worsening, defined as death from any cause, lung transplant, atrial septostomy, hospitalization for PAH for >24 h, or the worsening of symptoms. Over a median follow-up of 13.2 months, clinical worsening occurred in 10.6% of the sotatercept group versus 36.9% of the placebo group, corresponding to a 76% risk reduction (hazard ratio 0.24; 95% CI 0.13 to 0.43; *p* < 0.001). This study was also stopped early due to the superiority of the sotatercept arm and based on the positive interim efficacy data from the ZENITH trial [28].

Regarding safety, sotatercept demonstrated a consistent adverse event profile across clinical studies (Table 2). In the pivotal STELLAR trial, treatment-emergent adverse events were reported in 84.7% of patients receiving sotatercept and 87.5% of those receiving placebo, while serious adverse events occurred in 14.1% and 22.5%, respectively. The most relevant laboratory findings included an increase in hemoglobin and a decrease in platelet count. The mean hemoglobin level increased by 1.3 g/dL in the sotatercept group compared with 0.1 g/dL in the placebo group; an increase of >2 g/dL was observed in 12.3% of patients, and no increases > 4 g/dL were reported. These elevations were manageable with treatment interruptions or dose reductions and did not lead to treatment discontinuation. The mean platelet count decreased by 15.9 × 10^9^/L in the sotatercept arm compared with 1.1 × 10^9^/L in the placebo arm. No bleeding events associated with thrombocytopenia were observed. Adverse events occurring more frequently with sotatercept included epistaxis, gingival bleeding, dizziness, and telangiectasia [22]. The findings from earlier and subsequent trials (PULSAR, SPECTRA, ZENITH, and HYPERION) were consistent with the STELLAR results. Across studies, the same pattern of mild to moderate hemoglobin increases, moderate decreases in platelet count, and low rates of mucocutaneous bleeding was observed. Epistaxis, gingival bleeding, dizziness, and telangiectasia occurred more frequently under sotatercept treatment but were generally mild, non-serious, and reversible with dose modification or temporary interruption [21,22,24,25,28].

Several other studies on the efficacy and safety of sotatercept are currently ongoing. The SOTERIA study is an open-label, long-term follow-up study investigating the long-term efficacy and safety of sotatercept. Adult patients who have previously completed a clinical trial involving sotatercept are included. The interim results to date have confirmed a safety profile consistent with previous studies [29]. Notably, the results from SOTERIA show that most patients required some form of dose reduction or interval prolongation during long-term treatment.

Another notable study is the MOONBEAM study, a multicenter, open-label Phase 2 study that is the first to investigate the safety, tolerability, and pharmacokinetics of sotatercept in children. Patients between the ages of 1 and 18 with known PAH will be included and will receive 0.3 mg/kg sotatercept subcutaneously every 3 weeks for a period of 24 weeks. The primary endpoints primarily include pharmacokinetics but also the occurrence of adverse events such as thrombocytopenia and an increase in hemoglobin concentration. Secondary outcomes will measure efficacy. This study is expected to be completed in 2028 [20].

### 3.3. Limitations of Narrative Reviews

A key limitation of this work is its nature as a short-interval narrative review, which inherently restricts both the temporal and methodological scope of evidence synthesis [30]. The rapid evolution of pulmonary hypertension therapeutics means that relevant data published outside the search window may not be captured, and emerging pediatric sotatercept studies are only incompletely reflected. As a narrative rather than systematic review, study selection and interpretation are also subject to potential selection and reporting bias. Furthermore, the paucity of pediatric data necessitates extrapolation from adult studies, which limits the direct applicability of some conclusions to children.

## 4. Discussion

### 4.1. Differences and Similarities Between Pediatric and Adult PAH

To evaluate the potential use of sotatercept in pediatric PAH, it is essential to consider the similarities and differences between age groups. While PAH is defined the same way in both children and adults, its etiology differs. In infants, PPHN predominates, whereas in older children, IPAH, HPAH, and PAH-CHD are the most common [3]. Adults more frequently present with PAH associated with HIV, connective tissue diseases, schistosomiasis (in endemic regions) or porto-pulmonary hypertension and often have comorbidities such as diabetes, hypertension, chronic obstructive pulmonary disease or renal failure. In contrast, children and infants more often show chromosomal, genetic and syndromic anomalies [2].

Despite these differences, the underlying pathophysiological mechanisms appear similar. Histopathological studies show that vascular remodeling follows a similar sequence in both age groups: medial proliferation, intimal fibrosis, plexiform lesion formation, and thrombotic events [31]. Children, however, more often exhibit increased medial hypertrophy, less intimal fibrosis, and fewer plexiform lesions at diagnosis, with these patterns likely evolving with the age of the patient [32]. As mentioned above, the symptoms also differ to a certain extent between age groups. The most common ones, such as exertional dyspnea, fatigue, and syncope, occur in both adults and children. However, it has been observed that (pre-)syncope occurs more frequently in childhood, while right heart failure with peripheral edema is more frequent in adulthood. With increasing age, the clinical presentation in children progressively resembles that observed in adults [33].

### 4.2. Current Treatment Strategies and Extrapolation of Adult Data

Although pediatric and adult PAH differ in several aspects, they share substantial similarities. Therefore, many therapeutic strategies originally developed for adult patients are also applied to children. Extrapolation from adult data to pediatric populations is a common practice and is formally embedded in regulatory drug development pathways, particularly for rare diseases. Consequently, many drug approvals are at least partially based on extrapolated adult efficacy data [34]. This approach, however, largely reflects the limited evidence base for pediatric PAH therapy. Due to the low incidence of PAH in childhood, only a few randomized controlled trials have been conducted in this population [34]. Ethical considerations further limit the inclusion of children, given concerns regarding informed consent, risk exposure and long-term safety in a vulnerable population. As a result, current treatment algorithms rely heavily on adult data and expert opinion. Despite this, treatment has been shown to improve mortality rates in children just as effectively as in adults. Historically, untreated PAH led to death, with a 1-year survival rate of <37% [35]. With targeted therapies, survival rates increased to 86%, 80%, and 72% after 1, 3, and 5 years, respectively [14].

The drugs initially approved for adults have demonstrated efficacy in children. In a randomized controlled study, sildenafil, a PDE5 inhibitor, improved the maximal oxygen uptake by an average of 7.7%, in a dose-dependent manner in children [36]. Bosentan, an endothelin receptor antagonist, improved WHO functional class and hemodynamics in observational pediatric studies [37]. Calcium channel blockers benefited children with a positive vasoreactivity test in terms of improved hemodynamics and reduced mortality [38]. Prostaglandin derivatives, such as epoprostenol, proved to be equally effective in children and adults, significantly improving long-term survival in both groups [39]. The widespread off-label use of these agents in children reflects both their perceived clinical benefit and the lack of approved alternatives; in international registries, up to 80% of pediatric patients receive at least one PAH-targeted therapy [12].

Overall, the treatment effects observed in adult PAH can often, but not universally, be extrapolated to pediatric populations. Extrapolation is particularly challenging for therapies with novel mechanisms of action, as with sotatercept. Nevertheless, similar efficacy may be expected in pediatric patients, given the shared pathophysiological mechanisms of the disease. Owing to the expected anti-proliferative effects of sotatercept on SMCs, children might theoretically derive particular benefit from this treatment, as they are more prone to developing extensive medial proliferation. Notably, sotatercept study populations primarily consisted of adults with HPAH or IPAH, while patients with PAH associated with HIV infection, portal hypertension, or schistosomiasis were excluded. As these conditions are rare in children, this may facilitate more accurate extrapolation. However, PAH-CHD, which represents a substantial proportion of pediatric cases, remains underrepresented in adult trials. The parameters used in these studies—such a 6MWD, PVR, and maximal oxygen uptake—are also used in pediatric patients to assess disease severity [40]. However, some of these endpoints are not feasible in infants and young children, highlighting the broader challenge of lacking validated, age-appropriate outcome measures in pediatric PAH trials [41].

Nonetheless, differences in growth, organ maturation, and drug metabolism, together with the novelty of this drug class, limit extrapolation from adult data. To date, comparable pediatric short-term efficacy, tolerability and safety data are lacking. The MOONBEAM trial aims to explore these aspects, but it has several important limitations. As an open-label, single-arm study with an estimated enrolment of 42 participants, it will offer limited evidence regarding sotatercept’s efficacy. Although it will provide information on pharmacokinetics and common laboratory-based adverse events, it cannot evaluate rare side effects and long-term safety since it only has a study duration of 24 weeks [20]. This highlights the need for long-term follow-up and post-marketing registries.

### 4.3. Potential Pediatric-Specific Safety Concerns and Long-Term Considerations

The adverse events observed in adults are predominantly mild and do not interfere with therapy, resulting in a favorable risk–benefit profile. To date, however, mortality benefits have not been demonstrated, likely due to relatively small cohort sizes and a limited number of events [42]. Available data on long-term outcomes remain limited, and extended follow-up is required to fully characterize the safety profile of sotatercept. The ongoing SOTERIA study may provide further insight into long-term efficacy and safety in adult patients.

Beyond these clinical limitations, potential long-term safety concerns have been raised at the regulatory level. The FDA has highlighted a theoretical tumorigenic risk associated with the pharmacological blockade of the activin A signaling pathway [43]. Several studies have demonstrated the complex and context-dependent role of activin signaling in both tumorigenesis and tumor suppression. Activin A has been shown to function as a tumor suppressor in certain cell types, particularly during the early stages of tumor initiation [44,45]. This is further supported by the presence of genetic mutations disrupting the activin A signaling pathway in specific malignancies [46]. In contrast, elevated levels of activin A have been reported in other tumor types, underscoring its dual and tissue-specific function [47,48]. Accordingly, the pharmacological blockade of activin A signaling through sotatercept could interfere with these regulatory mechanisms and may theoretically contribute to tumorigenic processes. To date, however, no carcinogenic effects have been observed in preclinical short-term studies [43,49]. But given that tumor development is a long-term process, these findings do not exclude the possibility of oncogenic risk over extended periods. This consideration is particularly relevant in the pediatric population, where extended life expectancy and longer treatment durations may increase the likelihood of any potential tumorigenic effects that could manifest over time.

Sotatercept may also affect reproductive function, as activin A is a key regulator of the hypothalamic–pituitary–gonadal axis. At the pituitary level, activin stimulates the transcription and secretion of follicle-stimulating hormone (FSH), counterbalanced by inhibins and follistatin. A clinical study in postmenopausal women demonstrated that sotatercept decreases FSH levels [50]. FSH plays a central role in pubertal initiation and gonadal maturation in both sexes [51,52]. Preclinical studies have shown that sotatercept can alter reproductive organ histology, reduce fertility indices in rats, and delay sexual maturation in juvenile animals [43,49]. These findings indicate that the sustained inhibition of activin A signaling may affect pubertal timing, gonadal maturation and sexual development in pediatric patients. From a long-term perspective, potential effects on fertility are particularly important, as children and adolescents have their entire reproductive lifespan ahead. However, adult clinical trials have not systematically evaluated effects on fertility, likely due to the average patient age (~50 years) and therefore its limited relevance. Thus, these concerns remain largely theoretical, based on known biological functions and preclinical data.

Preclinical and clinical studies have also shown that sotatercept influences bone density, bone volume and bone mineralization [50,53,54]. In the pediatric setting, bone development is a highly dynamic process. Interference via activin A inhibition could therefore theoretically affect skeletal maturation and growth patterns in children.

These considerations underscore the need for careful risk–benefit assessment and the long-term monitoring of growth, skeletal maturation and pubertal development in pediatric patients treated with sotatercept.

**Figure 2:**
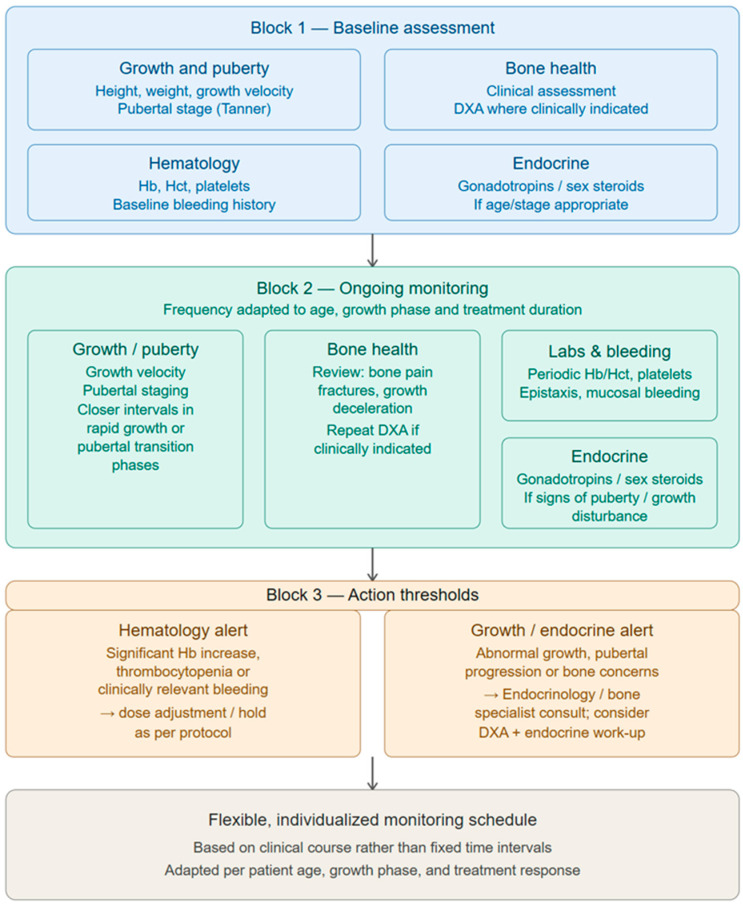
A suggestion for a principle-based monitoring framework for pediatric sotatercept therapy, integrating growth, pubertal and bone assessments with hematologic and endocrine surveillance while allowing for flexible, individualized follow-up intervals.

To ensure safety in pediatric patients receiving sotatercept—particularly considering potential effects on the hypothalamic–pituitary–gonadal axis and bone growth—a structured yet flexible monitoring framework is warranted. Baseline assessment should include anthropometric measurements (growth velocity, height/weight percentile, pubertal staging) and an evaluation of bone health, using dual-energy X-ray absorptiometry (DXA) where clinically indicated. During treatment, periodic reassessment should follow a principle-based approach tailored to growth stage and treatment duration rather than fixed intervals. Laboratory monitoring should encompass complete blood count with hemoglobin, hematocrit, and platelet counts, alongside surveillance for bleeding events. Endocrine evaluation (gonadotropins, sex steroids, thyroid function) should be performed if clinical signs suggest alterations in pubertal progression or growth (Figure 2). This pragmatic approach allows for the early detection of potential adverse effects while accommodating the heterogeneity of pediatric development and lack of data concerning the long-term risk factors in children.

### 4.4. The Role of Sotatercept in the Treatment of Persistent Pulmonary Hypertension of the Newborn (PPHN)

In the neonatal setting of persistent pulmonary hypertension of the newborn (PPHN), the potential role of sotatercept remains entirely theoretical, as no clinical data in this population are currently available. Given the unique pathophysiology of PPHN, including rapid and often reversible vascular remodeling in the immediate postnatal period, extrapolation from adult or older pediatric PAH data is not appropriate. At present, no guidelines or expert consensus statements recommend the use of sotatercept in PPHN, and its safety profile in preterm or term neonates is unknown. In particular, concerns regarding effects on vascular development, hematologic parameters, and organ maturation argue for a highly cautious stance. Accordingly, sotatercept cannot be suggested as a therapeutic option in PPHN outside of rigorously designed clinical trials, which are not yet available.

### 4.5. Call for Proactive Therapeutic Strategies in Pediatric PAH

The persistently poor outcomes in pediatric PAH underscore the need for a more proactive therapeutic strategy. Contemporary registry data indicating a 5-year survival of only around 70–75% in affected children highlight an unacceptably low benchmark for a life-limiting disease in an otherwise long-life-expectancy population. This unfavorable prognosis provides a clear ethical and clinical rationale to intensify the investigation of genuinely disease-modifying therapies rather than relying solely on incremental symptomatic approaches. In this context, sotatercept’s capacity to modulate BMPRII-related signaling and partially reverse pulmonary vascular remodeling in adults offers a compelling mechanistic rationale for its evaluation in children, in whom early intervention may help prevent progression to fixed, irreversible vascular changes. Moreover, in children with complex congenital heart disease who were previously deemed inoperable due to advanced pulmonary vascular disease, a sotatercept-mediated improvement in pulmonary hemodynamics could, at least conceptually, shift selected patients into an operable range and thereby reopen the window for definitive surgical repair. Accordingly, our discussion emphasizes that the field should not merely “wait” for the results of MOONBEAM but regard this trial as an essential first step within a broader strategy aimed at fundamentally improving the long-term outlook for children with PAH, including those with congenital heart disease previously considered beyond surgical rescue.

## 5. Conclusions

This review demonstrates that sotatercept, as a first-in-class TGF-β-targeted activin trap, pursues a clear disease-modifying approach in PAH, improving hemodynamics, exercise capacity, and right ventricular function in preclinical models and large adult trials (PULSAR, STELLAR, ZENITH, HYPERION). Shared pathomechanisms make extrapolation to pediatric PAH plausible, currently supported indirectly by adult data and the ongoing pediatric Phase 2 MOONBEAM trial. Substance-specific long-term risks particularly relevant for children—such as potential effects on tumorigenesis, puberty, fertility, and bone development—remain clinically unaddressed. Overall, sotatercept is positioned as a potentially transformative therapy for pediatric PAH, but broad application requires a systematic evaluation of efficacy and long-term safety in pediatric cohorts.

## Figures and Tables

**Table 1:** Comparison of key characteristics of four sotatercept randomized controlled trials (PAH—pulmonary arterial hypertension, WHO FC—World Health Organisation functional class, RCT—randomized control trial, SC—subcutaneous, 6MWD—6 min walk distance, PVR—pulmonary vascular resistance, LSM—least squares mean).

Category	PULSAR	STELLAR	ZENITH	HYPERION
**MAIN INVESTIGATION**	Safety and efficacy of sotatercept in patients with PAH	Safety and efficacy of sotatercept in patients with PAH	Sotatercept in advanced PAH (WHO FC III/IV) focusing on clinical worsening	Sotatercept in newly diagnosed WHO FC II/III PAH (<12 months) at medium to high risk
**STUDY DESIGN**	Phase 2 RCT	Phase 3 RCT	Phase 3 RCT	Phase 3 RCT
**POPULATION**	PAH in WHO functional class II/III	PAH of WHO functional class II/III	High-risk PAH	Newly diagnosed PAH (<1 year)
**MAIN INCLUSION** **CRITERIA**	1. Age ≥ 18 2. Confirmed WHO Group 1 PAH 3. WHO FC II or III 4. Stable background PAH therapy for ≥90 days	1. Age ≥ 18 2. Confirmed WHO Group 1 PAH 3. WHO FC II or III 4. Stable background PAH therapy for ≥90 days	1. Age ≥ 18 2. Symptomatic WHO Group 1 PAH 3. WHO FC III or IV (high risk) 4. REVEAL Lite 2 risk score ≥ 9 5. Stable double or triple therapy for ≥30 days	1. Age ≥ 18 2. Confirmed WHO Group 1 PAH 3. WHO FC II or III 4. PAH diagnosis received <1 year earlier 5. REVEAL Lite 2 risk score ≥ 6 or COMPERA 2.0 risk score ≥ 2 6. Stable double or triple therapy for ≥90 days
**NUMBER OF PATIENTS**	106	323	172	320
**TREATMENT DURATION**	24 weeks	24 weeks	Stopped early, ~10.6 months (median)	Stopped early, ~13.2 months (median)
**DOSING**	SC sotatercept at a dose of 0.3 mg/kg or 0.7 mg/kg every 3 weeks	SC sotatercept at a starting dose of 0.3 mg/kg and target dose of 0.7 mg/kg every 21 days	SC sotatercept at a starting dose of 0.3 mg/kg and target dose of 0.7 mg/kg every 21 days	SC sotatercept at a starting dose of 0.3 mg/kg and target dose of 0.7 mg/kg every 21 days
**CLASSIFICATION OF PAH, %**				
**IDIOPATHIC**	58	58.5	50.0	59.4
**HERITABLE**	16	18.3	10.5	5.9
**ASSOCIATED WITH** **CTD**	17	14.9	27.9	30.3
**OTHER**	10	8.4	11.6	4.4
**MEAN AGE, YR**	48.3 ± 14.3	47.9 ± 14.8	54.4 ± 14.3	56.2 ± 16.4
**SEX (FEMALE), %**	87	79.3	76.7	72.5
**PRIMARY ENDPOINT**	Change in PVR	Change in 6MWD	Clinical worsening as a composite of death, transplant, or hospitalization	Clinical worsening as a composite of death, transplant, hospitalization, etc.
**OUTCOME**	Reduction in PVR vs. placebo; LSM change −162 dyn·s·cm^−5^ (0.3 mg/kg) and −256 dyn·s·cm^−5^ (0.7 mg/kg) vs. −16 dyn·s·cm^−5^ with placebo	6MWD increase at 24 weeks of +34 m vs. +1 m with placebo; mean between-group difference about +41 m	Events in 17.4% with sotatercept vs. 54.7% with placebo; hazard ratio 0.24 (≈76% risk reduction); trial stopped early due to superiority of sotatercept	Events in 10.6% with sotatercept vs. 36.9% with placebo; hazard ratio 0.24 (≈76% risk reduction); stopped early after positive interim data

**Table 2:** Comparison of adverse events in four sotatercept randomized controlled trials.

Category	PULSAR	STELLAR	ZENITH	HYPERION
**ADVERSE EVENTS (AES), %**				
**PLACEBO**	88	87.5	96.5	90.0
**SOTATERCEPT**	91 (0.3 mg/kg), 81 (0.7 mg/kg)	84.7	98.8	89.4
**SOTATERCEPT-SPECIFIC AE OF INTEREST (%)**	Thrombocytopenia (12) Hemoglobin increase (17)	Thrombocytopenia (6.1) Hemoglobin increase (5.5) Bleeding event (21.5) Epistaxis (20.2) Increased blood pressure (3.7) Telangiectasia (10.4) Dizziness (14.7)	Hemoglobin increase (12.8) Bleeding event (62.8) Epistaxis (44.2) Gingival bleeding (10.5) Telangiectasia (25.6)	Hemoglobin increase (11.2) Bleeding event (41.2) Epistaxis (31.9) Increased blood pressure (6.9) Telangiectasia (26.2)
**SERIOUS AE, %**				
**PLACEBO**	9	22.5	64.0	28.1
**SOTATERCEPT**	6 (0.3 mg/kg), 24 (0.7 mg/kg)	14.1	53.5	24.4

## Data Availability

No new data were created or analyzed in this study.

## Abbreviations

The following abbreviations are used in this manuscript:

6MWD	6 min walk distance
ACTRIIA	activin receptor type IIA
BMPR2	bone morphogenetic protein receptor-2
CI	confidence interval
EC	endothelial cell
EMA	European Medicines Agency
FDA	U.S. Food and Drug Administration
FSH	follicle-stimulating hormone
GDF	growth differentiation factor
HPAH	heritable pulmonary arterial hypertension
IPAH	idiopathic pulmonary arterial hypertension
LSM	least squares mean
mPAP	mean pulmonary artery pressure
PAH	pulmonary arterial hypertension
PAH-CHD	pulmonary arterial hypertension associated with congenital heart disease
PAWP	pulmonary artery wedge pressure
PDE5	phosphodiesterase type 5
PH	pulmonary hypertension
PPHN	persistent pulmonary hypertension of the newborn
PVR	pulmonary vascular resistance
RV	right ventricular
SMC	smooth muscle cell
TGF	transforming growth factor
WU	Wood unit
